# Reproductive decision-making following the diagnosis of an inherited metabolic disorder via newborn screening in Japan: a qualitative study

**DOI:** 10.3389/frph.2023.1098464

**Published:** 2023-05-18

**Authors:** Kana Hiromoto, Masakazu Nishigaki, Shinji Kosugi, Takahiro Yamada

**Affiliations:** ^1^ Department of Medical Ethics and Medical Genetics, Kyoto University School of Public Health, Kyoto, Japan; ^2^ Department of Human Health Sciences, School of Medicine, Kyoto University, Kyoto, Japan

**Keywords:** genetic counseling, genetic diseases, newborn screening, decision making, family planning

## Abstract

**Introduction:**

The aim of the study was to describe the factors influencing the reproductive decision-making of carrier parents after the diagnosis of an inherited metabolic disorder in newborn screening in Japan.

**Methods:**

We conducted a semi-structured interview with 12 parents and analyzed data based on content analysis methodology.

**Results:**

We identified 11 factors, including personal evaluation of recurrence risk, understanding of hereditary phenomena, concerns and desires for future planned children, concerns for older siblings, perceptions of diseases, degree of acceptance and denial of diseases, the opinions of others on having another child, optimism/faith in positive outcomes, self-evaluation of parental capability, factors unrelated to the disease, and the “right” time to expand the family.

**Discussion:**

Perceptions and acceptance of disease are both important factors in reproductive decision-making, though these factors fluctuate continuously during the childbearing period. Therefore, effective reproductive genetic counseling will be considerate of the parents' fluctuating perceptions on reproduction. To ensure that the decision-making process is for the benefit of the parents and future children, long-term involvement of health care professionals is needed to assess the client's acceptance of the disease and their understanding of genetic phenomena and recurrence rates.

## 1. Introduction

Congenital inherited metabolic disorders (IMDs) can have serious consequences for the affected infants. However, some IMDs are treatable and/or preventable if they are detected in the presymptomatic period by the newborn screening program (NBS) (1).

In Japan, the NBS was established as a public health program in 1977, and is now available nationwide (2). Since its establishment, the number of patients diagnosed with IMDs has increased considerably. More than 20 diseases are covered by the program, with 800–1,000 people diagnosed annually (3, 4).

Diagnosis of IMDs in infants also reveals the genetic background of the parents, given that most IMDs are autosomal recessive. However, most parents are unaware of being genetically tested, which can cause additional confusion or anxiety in parents who have already received unexpected news about their child. Additionally, many parents are surprised by the diagnosis of their child as the clinical symptoms can often be pervasive. Evidence has shown that most parents are overwhelmed not only by the news of their child being affected, but also by the unexpected genetic realities (5). Carrier parents often lack an understanding of the implications and potential for recurrence in future offspring (6).

Family planning is therefore a major topic in the genetic counseling of parents with children diagnosed with autosomal-recessive IMDs by NBS. Gee et al. (7). reported that previous studies of family planning in carrier parents of hereditary disorders (8, 9) focused mainly on the parents' perceptions of prenatal diagnosis or selective abortions. However, preventable and/or treatable diseases, including NBS target diseases, are less eligible for prenatal diagnosis in Japan (10). Additionally, artificial abortion based on fetal indications is not permitted by the Maternal Health Act, since selective abortion is not regarded as part of reproductive autonomy (11). Moreover, several patient and disability support groups in Japan oppose prenatal diagnosis and selective abortion (12). Therefore, the process of reproductive decision-making of parents following the birth of an affected child should be discussed without the context of prenatal testing in Japan. Thus, in-depth discussions regarding reproductive decision-making after NBS are required in the context of Japanese law, ethics, and culture. The objective of our study was to identify the factors influencing the consideration of Japanese parents for further pregnancy after the birth of a child diagnosed by the NBS with an IMD.

## 2. Materials and methods

In this study, we conducted a secondary analysis of interview data collected in a previous study investigating the emotional reaction of parents following their child's diagnosis with an IMD (6).

### 2.1. Participant recruitment

The study participants (*n* = 12) were mothers or fathers of children diagnosed with an IMD by the NBS, all of whom were informed about recurrence rate (25%) by a pediatrician. The final number of participants was based on the minimum number required for statistical relevance (13). Participant exclusion criteria included: (1) the child was diagnosed with a non-hereditary congenital hypothyroidism, (2) the genetic characteristics of the IMD are unknown, and (3) the case was considered unsuitable at the authors' discretion, e.g., a case where the interview could not be completed due to illness.

The study participants were recruited through two groups, the Phenylketonuria (PKU) Parents' Association (https://www.japan-pku.net) and Hidamari Tanpopo (Dandelion in sunny place) (http://hidamari-tanpopo.main.jp), both of which are patient/family associations for organic and fatty acid metabolism disorders. Participants were recruited through: (1) online recruitment guidance sent directly to the members of the groups, (2) onsite recruitment at member meetings, (3) attachment of participant recruitment guidance to newsletters, and (4) advertisement of the study on the groups' websites. Written informed consent was obtained from all participants. This study was approved by the Ethics Committee of the Kyoto University Graduate School and Faculty of Medicine (C1442).

### 2.2. Data collection

The data for this qualitative study were collected from September to December 2019 through semi-structured one-on-one interviews conducted over Skype (https://www.skype.com/ja/ or by telephone. The following five topics were the focus of the interview: (1) perceptions of the disease, (2) emotions evoked by diagnosis, (3) emotions evoked by disclosure of the recurrence rate in siblings, (4) factors to consider in planning for another child, and (5) support needed by parents. These topics were selected based on previous studies (14–16). Given the objectives of the present study, we focused on the narratives relating to reproductive decision-making (topic 4), which were complemented with the perceptions gained from topics 1–3 and 5.

All interviews were conducted by K.H. and included audio recordings and verbatim transcripts. Data saturation was confirmed when no new decision-making factors were generated for at least two interview sessions.

### 2.3. Data analysis

The relevant characteristics for each participant are presented in Table 1. Interview data were analyzed using a content analysis method for factor extraction (17). The analysis was performed using Microsoft Excel. The credibility of each factor was verified by researcher triangulation. The analyses were conducted by K.H. under the supervision of T.Y. The analysis was performed using Microsoft Excel. The credibility of each factor was verified by researcher triangulation. The analyses were conducted by K.H. under the supervision of T.Y.

**Table 1:** Participants’ attributes.

Number	Parental age	Participant's Gender	Age of affected child	Type of diseases	Symptoms	Treatment option	Genetic testing	Carriertesting	Birth order^a^	Interview method
1	48	F	18	PKU^b^	×	Dietary adjustment	○	○	●□	Skype
2	48	F	8	PKU	×	Dietary adjustment	×	×	□●	Skype
3	41	F	4	PKU	×	Dietary adjustment	×	×	□●	Skype
4	41	M	4	PKU	×	Dietary adjustment	×	×	○●#□#	Skype
5	38	F	4	PKU	×	Dietary adjustment	×	×	▪□	Skype
6	43	F	9	PKU	×	Dietary adjustment	×	×	○▪	Skype
7	35	F	0	Propionic acidemia	×	Medication	○	×	□□▪	Telephone
8	32	F	1	Methylmalonic acidemia	×	Medication	○	×	●	Skype
9	35	F	1	VLCAD^c^ deficiency	×	Dietary adjustment	○	×	□▪	Skype
10	36	F	8	Homocystinuria	×	Liver transplantation, Medication, Dietary adjustment	×	×	□●□	Skype
11	51	M	3	Methylmalonic acidemia	Poor feeding, Repeated vomiting	Dietary adjustment	○	×	●	Telephone
12	43	F	11, 8	PKU	×	Dietary adjustment	○	×	●●	Telephone

^a^ □, Indicates a healthy boy; ▪, indicates an affected boy; ○, indicates a healthy girl; and ●, indicates an affected girl. Siblings marked with # are dizygotic twins.

^b^ PKU, phenylketonuria.

^c^ VLCAD, very long-chain-acyl-dehydrogenase.

During initial coding, every meaning unit was encoded after thorough examination of the transcripts. These codes were then assigned to subcategories clustered within decision-making factors.

## 3. Results

Participant suitability was determined with a pre-interview questionnaire. None of the participants had a prenatal diagnosis of an IMD. Nine participants were interviewed on Skype, and the remaining three over the phone. The average length of the interviews was 62 min (range: 37–79 min).

Through the interviews, we generated 11 decision-making factors comprising 42 subcategories (Table 2). We discuss each of these factors below, along with personal accounts from the participants.

**Table 2:** Categories of factors involved in reproductive decision-making for parents of children affected by IMDa.

Categories	Subcategories
[1] Personal evaluation of recurrence risk	Influences from practitioners
	Experience-based belief
	Irrational belief resulting from unscientific evaluation
	Knowledge of recurrence phenomena
	Illogical evaluation
[2] Understanding of hereditary phenomena	Adequate/inadequate understanding of the cause of the disease
	Knowledge level on genetics
[3] Concerns and desire for future planned children	Chances of being affected with IMD^a^
	Chances of being affected with other disorders
	Health management efforts
	Urge to have more children
[4] Concerns for older siblings	The prospect of affected children having affected siblings as peers, or not being left alone after parents pass away
	The chances of healthy children being upset or having to sacrifice themselves as caregivers for affected siblings
[5] Perceptions of diseases	Influences of online information
	Influences of other people's attitudes
	Acquisition of sense of control along with better understanding of the disease
	Physical condition of the affected children
	Parents’ state of mind
	The treatable/preventable characteristics of the disease
	Invisibility of the disease
[6] Degree of acceptance of diseases	Growth and development of the affected child
	Influences of other people
	Recovery from panic over time
	Quality of information on the disease
	Possible future treatment options
	Parental self-efficacy
	Good communication with health professionals
[7] Other people's opinions on having another child	Supportive comments from fellow parents
	Family members’ opinions
	Expert opinions from health professionals
[8] Belief in good luck	Nonevidence-based optimistic conviction about having a healthy child
[9] Self-evaluation of parental capability	Self-evaluation of parental capacity
	Financial capabilities
[10] Factors unrelated to the disease	Ideal family size
	Gender preference
	Maternal age
	Parental physical condition
	Child raising philosophy
[11] The “right” time to expand family	Achieving the optimal adjustment to life with affected children
	Health management skills
	Ideal age gap between siblings
	Intuition of having a healthy child

^a^ IMD, inherited metabolic disorders.

### 3.1. Factor 1: personal evaluation of recurrence risk

A participant's personal evaluation of recurrence risk was influenced by the people around them, including communication with their doctors, encounters with fellow patients, experience-based belief or irrational belief resulting from unscientific evaluation, and inadequate knowledge of recurrence phenomena resulting in illogical evaluation. As expected, health professionals played a critical role. When parents felt well-informed, they were less anxious and more motivated for parenthood. Some participants were unable to interpret the implications of disease recurrence rate due to a lack of knowledge and could not use this as a criterion in their decision-making.

“Talking with fellow parents, I've come to realize having affected children in a row isn't as common as I worried. That makes me feel relieved to try for the next pregnancy.” (12)

“Theoretically speaking, the recurrence risk is 25%. But for us parents, the odds are always half and half, you know what I mean. Affected or not. That's all.” (3)

“I decided the next one I was expecting would naturally be affected with PKU. To be honest, I was very scared of expecting a healthy baby for I didn't believe I could bear the shock of ending up with the opposite.” (1)

### 3.2. Factor 2: understanding of hereditary phenomena

An adequate understanding of the causes of an IMD relieved parents from any sense of guilt and provided positive perspectives toward their reproductive decision, while an inadequate understanding had the opposite effect. Knowledge of genetics, especially regarding the non-symptomatic carriage of genes related to autosomal-recessive diseases, also influenced reproductive decision-making. Parents that were naive to the field of genetics were either irrationally convinced that they could not be carriers or were unnecessarily pessimistic of having an affected child.

“Talking about heredity, no one should be blamed. I mean, there's no point in searching for the culprit when it really doesn't exist. It just can't be helped. Nobody is wrong here.” (1)

“Honestly, I feel sorry and blame myself constantly, thinking it's all my fault. I might be wrong but cannot stop feeling this way.” (2)

“Learning of the chances of me being a carrier, I thought maybe I was the one who needed treatments, not my kid.” (8)

“The idea of being a carrier never occurred to me. I have always been doing fine, just as fine as many other healthy people out there.” (5)

### 3.3. Factor 3: concerns and desires for future planned children

Many participants hesitated in planning another pregnancy due to concerns about having another affected child, while others felt impelled to have more children. The likelihood of being affected with an IMD was a major determinant of the desire to have another child. Experience in disease management (i.e., nutritional management) moderated the degree of concern. Participants also expressed a sense of guilt for wanting another child, knowing that it may be affected. Some parents showed concern for their next child being affected with other disorders than metabolic disorders, which emerged from their encounters with children having several different congenital disorders. Participants were also concerned about effective health management and maintaining the quality of life for affected children. Some parents expected healthy siblings to be primary caregivers later in life, a responsibility that they deemed would not be very challenging as metabolic disorders are generally treatable/preventable. Participants whose children were all affected by IMDs showed a desire for healthy children.

“After the experience with an affected kid, I came to think about other diseases and realized that there are an awful lot of kinds of illness.” (1)

“I simply wanted to be a mom again. I wanted to have another baby and take it in my own hands.” (10)

“In fact, I just wanted to raise a normal kid as well.” (1)

“As for phenylketonuria, which doesn't require much health management, it wouldn't be challenging for younger unaffected siblings to have an affected sister if I were to have further children.” (12)

### 3.4. Factor 4: concerns for older siblings

Some parents of affected children recognized the advantages of having another child: he/she could support the affected child and, if they were also affected, they would share a more intimate understanding of life with the disease. However, participants with both affected and healthy children showed concerns that the healthy siblings could be upset by another affected sibling or they might sacrifice their own quality of life as caregivers for the affected siblings.

“I started to think having another kid with the same disease as the older one isn't bad at all. They can share a lot, supporting each other when needed.” (6)

“My older (healthy) kids would be quite upset to learn that they have two affected siblings in a row. l thought the idea of genetic defects might break their heart.” (7)

### 3.5. Factor 5: perceptions of diseases

The influence of online information was a major factor influencing perceptions of diseases. Most participants obtained information or treatment options when they learned of their child having a lifelong disease. However, their perceptions of the diseases were likely to be influenced by the attitudes of others, a sense of control along with a better understanding of the disease, physical condition of the affected children, or the parent's state of mind.

While some parents were optimistic knowing that disease onset was avoidable, others were nervous because some IMDs would make normal life impossible. If parents were faced with a more serious disorder, their desire for another child was reduced.

“Searching online, it's more often than not that those articles you'd come across focus only on unusual, severe cases. At first, I really didn't get the right picture of what was going on with my kid and I was extremely anxious to receive the final test result because I had read about and heard of cases where intellectual delay took place.” (7)

“Compared to those kids with hyper allergies, it's way easier for us to cope with as it's not something acute.” (6)

“The kids are growing all fine and I know they'll be doing just fine like many other kids as long as we stick to the right food choices. This makes me feel much more positive.” (4)

“Honestly, I guess we're lucky enough because ours were detectable in the NBS unlike many other illnesses. I have nothing to complain about for it to be found and treated.” (6)

### 3.6. Factor 6: degree of acceptance of diseases

Most parents found it difficult to accept the diagnosis despite significant evidence. Similar to perceptions of diseases, the degree of acceptance of diseases varied widely. The acceptance status of the disease was affected by the growth and development of the affected child; influences from others, including encounters with people from patient associations; communication with healthcare professionals; and the perceptions of partners. In most cases, parents were confused by the notification of the disease. However, with time to consider the disease, which could be provided by hospitalization with the affected child, some parents learned to accept the disease.

The availability and quality of information were important determinants of acceptance of diseases. Lack of accurate information often led to irrational attitudes, including excessive fear or panic, while high-quality information contributed to a better understanding and acceptance of the disease. Acceptance enhanced the parent's motivation for continued management efforts, such as dietary adjustments. Some parents accepted their child's disease with optimism for future treatment options and/or improved parental efficacy in disease management. Moreover, some accepted the disease through communication with healthcare professionals.

“After two and a half years with the first affected kid, I kind of started getting used to it and that made me think I could afford another baby. I knew I would be doing good enough as a mom.” (12)

“Watching my kids growing fine does make a very big difference, I guess. That makes me feel really positive.” (9)

“Knowing I was able to save food options for my kid calmed me down and now I'm feeling much easier as there always are food choices to choose from. We are not as limited as some may think we are.” (1)

### 3.7. Factor 7: the opinions of others on having another child

Remarks from others influenced the decision of participants to have another child after an affected one. Supportive comments from parents who decided to have another child or who had multiple affected children provided encouragement for further pregnancy. However, the opinions of family members and experts could discourage further pregnancy.

“When you already have an affected one, having one more with the same needs isn't as hard as it may look. With the first one, you have learned pretty much about how to deal with, say, school, schoolteachers, and lunches.” (12)

“My husband tells me having more kids after an affected one doesn't sound great at all. I really don't have any idea what is the right decision for us.” (7)

“My physician told me everyone on the team would do their very best to help us with the next kid if that was what I really wanted. Their attitudes encouraged me indeed.” (11)

### 3.8. Factor 8: optimism/faith in positive outcomes

Some participants were optimistic about their prospects of having healthy children in future pregnancies, with no scientific basis. This faith in positive outcomes naturally contributed to their decision to try for another pregnancy.

“I was just convinced that the next one would be perfectly alright just like many other kids. I might have been wrong but honestly that was what I thought.” (1)

“I don't know if anyone would understand me, but I just knew I was going to have a baby free of PKU.” (12)

### 3.9. Factor 9: self-evaluation of parental capability

Another factor identified in the interviews was the confidence parents had in their parental capabilities. Higher rates of self-evaluation of parental capacity were associated with a stronger desire to have another child, while lower rates had the opposite effect.

The evaluation included financial security and, not surprisingly, parents who rated their financial security as high had a stronger desire for another child. Dietary treatments often require specially formulated foods that are relatively expensive, with some participants finding these foods unaffordable.

“I just thought having another kid was something far beyond us.” (4)

“Given the financial difficulties we would be facing, we decided not to go for another pregnancy. With more than one affected kid, the monetary cost of medical foods would make it impossible for us to make ends meet.” (1)

### 3.10. Factor 10: factors unrelated to the disease

The ideal family size, gender preference, maternal age, and parental physical condition were important determinants in reproductive decision-making. Other factors included the child-rearing philosophy followed before having children with special needs. Some participants had a philosophy of “accepting anything that happened to their children” and others believed having siblings would be good regardless of their needs.

“I wasn't young anymore and I wanted to hurry to have another baby. I couldn't afford the wait.” (12)

“For us, two was always the ideal number of kids and that didn't change after the birth of affected baby.” (2)

“After all, (regardless of child's condition) being a parent means going through the whole process to accept whatever that is going on in our life.” (1)

### 3.11. Factor 11: the “right” time to expand the family

Participants felt that the ideal time for having another child was under the following conditions: full adjustment to life with affected children, health management skills have been acquired, ideal age gap between siblings, and intuition of having a healthy child. The latter is not an illogical evaluation coming from naivety nor is it faith in positive outcomes, but refers to a more spiritual intuition.

“I found myself thinking about having another baby after I had gone through the surreal and overwhelming period, coming back to the ‘real’ life, you know.” (5)

“I knew I needed to learn how to cope with it before the next baby comes in our life. I decided to wait until the kid was old enough to have the same meals with adults. I wanted to get the whole picture of it, like knowing how much food limitation would take place.” (8)

“My intuition or my mother's instinct was telling me it was the perfect time to have the healthy one.” (10)

## 4. Discussion

### 4.1. Factors that influence the decision for further pregnancy

In this study, we identified factors involved in the reproductive decision-making of parents with one or more children diagnosed with IMDs in Japan. The major factors determined in this study were similar to those of previous studies of people with hereditary tumor or neuromuscular diseases (18), which suggests that these studies were at the same level of abstraction. However, unlike many patients affected with other hereditary disorders, the participants in our study were completely unaware that they were carriers and did not expect an IMD diagnosis. As expected, they often did not have sufficient knowledge on recurrence rate and genetic phenomena, neither of which had been relevant until they received the NBS results.

Although life with IMD-diagnosed children can be difficult, early intervention can prevent disease onset and parents can achieve effective disease treatment with dietary management. These positive perspectives are specific to IMDs.

This study revealed that parents' personal evaluations of recurrence rate, understanding of hereditary phenomena, perception of diseases, and level of acceptance/denial of diseases are greatly influenced by the opinions of medical professionals. Werner-Lin et al. (16), using Cox's Interaction Model of Client Health Behavior, found that the client–health professional interaction is among the key factors in reproductive decision-making. Our results indicate that dynamic or changeable factors, such as perception of diseases (greatly influenced by medical experts), were among the most important factors in parental decision-making along with practical factors, such as maternal age. Consequently, healthcare professionals, including genetic counselors, play a crucial role in a client's reproductive decision-making. In genetic counseling for further pregnancy, careful attention should be paid to the clients' opinions and the factors involved in their reproductive decision-making, and close examination of each factor is required. This approach will empower the client in making an optimal decision according to available information.

Naturally, general reproductive factors such as ideal family size, maternal age, and parental physical condition affect the reproductive decision-making of most parents. Some of our participants were too overwhelmed to contemplate IMDs as an influential factor for reproductive decision-making. Considering hereditary diseases, health professionals often focus on heredity first (13), though attention should also be paid to these nonmedical factors.

### 4.2. Timing of support for parents considering further pregnancy

In this study, confusion following diagnosis by the NBS or receipt of carrier screening results did not emerge among the factors involved in reproductive decision-making. This is expected as active decision-making cannot be performed before the parents’ psychosocial adjustment to their child's condition. Through the process of acceptance, adjustment, and improvement of health management skills, they may recover, return to regular life, and proceed with reproductive decision-making.

There is no “perfect time for everybody” to have another child; this varies between individuals and depends on both dynamic factors, such as financial security or disease management skills, and the parent's physical condition, such as reduced fertility resulting from advanced maternal age. Therefore, counselors must consider these dynamic changes to meet their clients' needs accordingly. In Japan, children with IMDs are usually required to make a hospital visit once per month for their condition to be monitored. Genetic counselors should take advantage of these opportunities to establish a relationship based on trust and understanding and relate to the family's needs in a timely manner.

### 4.3. Conclusion

Perceptions and acceptance of disease are both important factors in reproductive decision-making, though these factors fluctuate continuously during the childbearing period. Therefore, effective reproductive genetic counseling will be considerate of the parents' fluctuating perceptions on reproduction.

### 4.4. Practice implications

Parents can easily be confused by the sudden announcement of a hereditary disease. However, with enough time to understand the disease and consider their child's needs, parents can learn to accept the disease. In this regard, it is important for counselors to follow up with the clients and promote their acceptance of the disease. Understanding of disease management and personal capabilities would also promote their positive perception of the disease and consideration of further pregnancy. Providing updated information empowers parents to cope with the disease and make informed reproductive decision-making. To ensure that the decision-making process is for the benefit of the parents and future children, long-term involvement of health care professionals is needed to assess the client's acceptance of the disease and their understanding of genetic phenomena and recurrence rates.

### 4.5. Study limitations

We recruited participants online to increase accessibility and maximize the number of candidates. This recruitment method could bias our participants to an information-literate population. Additionally, all participants were members of the patient support associations and showed keen interest in our study, which could imply a proactive attitude toward reproductive decision-making.

## Data Availability

The original contributions presented in the study are included in the article, further inquiries can be directed to the corresponding author.
